# Rare and low-frequency coding genetic variants contribute to pediatric-onset multiple sclerosis

**DOI:** 10.1177/13524585221150736

**Published:** 2023-02-08

**Authors:** Mary K Horton, Joan E Shim, Amelia Wallace, Jennifer S Graves, Gregory Aaen, Benjamin Greenberg, Soe Mar, Yolanda Wheeler, Bianca Weinstock-Guttman, Amy Waldman, Teri Schreiner, Moses Rodriguez, Jan-Mendelt Tillema, Tanuja Chitnis, Lauren Krupp, T Charles Casper, Mary Rensel, Janace Hart, Hong L Quach, Diana L Quach, Catherine Schaefer, Emmanuelle Waubant, Lisa F Barcellos

**Affiliations:** Division of Epidemiology, School of Public Health, University of California, Berkeley, CA, USA/Center for Computational Biology, College of Engineering, University of California, Berkeley, CA, USA; Division of Epidemiology, School of Public Health, University of California, Berkeley, CA, USA; Division of Epidemiology, School of Public Health, University of California, Berkeley, CA, USA/Department of Human Genetics, University of Utah, Salt Lake City, UT, USA; Department of Neurosciences, School of Medicine, University of California, San Diego, CA, USA/Department of Neurology, University of California, San Francisco, CA, USA; Pediatric MS Center, Loma Linda University Children’s Hospital, San Bernardino, CA, USA; Department of Neurology, University of Texas Southwestern, Dallas, TX, USA; Pediatric-Onset Demyelinating Diseases and Autoimmune Encephalitis Center, St. Louis Children’s Hospital, Washington University School of Medicine, St. Louis, MO, USA; Alabama Center for Pediatric-Onset Demyelinating Disease, Children’s Hospital of Alabama, Birmingham, AL, USA; Pediatric Multiple Sclerosis Center, Jacobs Neurological Institute, SUNY Buffalo, NY, USA; Division of Neurology, Children’s Hospital of Philadelphia, Philadelphia, PA, USA; Children’s Hospital Colorado, University of Colorado, Denver, CO, USA; Mayo Clinic’s Pediatric Multiple Sclerosis Center, Rochester, MN, USA; Mayo Clinic’s Pediatric Multiple Sclerosis Center, Rochester, MN, USA; Partners Pediatric Multiple Sclerosis Center, Massachusetts General Hospital for Children, Boston, MA, USA; Lourie Center for Pediatric Multiple Sclerosis, Stony Brook Children’s Hospital, Stony Brook, NY, USA; Department of Pediatrics, University of Utah School of Medicine, Salt Lake City, UT, USA; Mellen Center, Cleveland Clinic, Cleveland, OH, USA; Regional Pediatric MS Center, Neurology, University of California, San Francisco, CA, USA; Division of Epidemiology, School of Public Health, University of California, Berkeley, CA, USA/Center for Computational Biology, College of Engineering, University of California, Berkeley, CA, USA; Division of Epidemiology, School of Public Health, University of California, Berkeley, CA, USA/Center for Computational Biology, College of Engineering, University of California, Berkeley, CA, USA; Kaiser Permanente Division of Research, Oakland, CA, USA; Department of Neurology, University of California, San Francisco, CA, USA; Division of Epidemiology, School of Public Health, University of California, Berkeley, CA, USA/Center for Computational Biology, College of Engineering, University of California, Berkeley, CA, USA/Kaiser Permanente Division of Research, Oakland, CA, USA

**Keywords:** Multiple sclerosis, pediatric-onset, rare variants, POMS, GWAS

## Abstract

**Background:**

Rare genetic variants are emerging as important contributors to the heritability of multiple sclerosis (MS). Whether rare variants also contribute to pediatric-onset multiple sclerosis (POMS) is unknown.

**Objective:**

To test whether genes harboring rare variants associated with adult-onset MS risk (*PRF1, PRKRA, NLRP8*, and *HDAC7*) and 52 major histocompatibility complex (MHC) genes are associated with POMS.

**Methods:**

We analyzed DNA samples from 330 POMS cases and 306 controls from the US Network of Pediatric MS Centers and Kaiser Permanente Northern California for which Illumina ExomeChip genotypes were available. Using the gene-based method “SKAT-O,” we tested the association between candidate genes and POMS risk.

**Results:**

After correction for multiple comparisons, one adult-onset MS gene (*PRF1, p* = 2.70 × 10^−3^) and two MHC genes (*BRD2, p* *=* 5.89 × 10^−5^ and *AGER, p* *=* 7.96 × 10^−5^) were significantly associated with POMS. Results suggest these are independent of HLA-DRB1*1501.

**Conclusion:**

Findings support a role for rare coding variants in POMS susceptibility. In particular, rare minor alleles within *PRF1* were more common among individuals with POMS compared to controls while the opposite was true for rare variants within significant MHC genes, *BRD2* and *AGER*. These genes would not have been identified by common variant studies, emphasizing the merits of investigating rare genetic variation in complex diseases.

## Introduction

There is substantial evidence for genetic factors contributing to adult-onset multiple sclerosis (MS) risk. The strongest genetic variants associated with MS are within the major histocompatibility complex (MHC), a genomic region on chromosome 6 involved in adaptive immune responses.^1,2^ Genome-wide associated studies (GWAS) of common variants (minor allele frequency (MAF) > 5%) have identified 32 independent loci in the MHC and >200 non-MHC variants that contribute to MS risk.^3^ However, these common variants only account for 20% of MS heritability.^4^ One emerging theme in genetic studies has been the importance of rare (MAF < 1%) and low-frequency (MAF < 5%) variants in disease heritability. Common variants included in GWAS often have small effect sizes and contribute a small-to-moderate portion of heritability. On the contrary, rare or low-frequency variants can result in large effect sizes with a surprisingly high contribution to heritability.^5^ The largest study to investigate the effects of rare and low-frequency variants on MS risk determined these variants explain an additional 5% of heritable risk of MS among adults.^4^ Significant associations were identified between MS risk and seven low-frequency coding variants in six genes outside the extended MHC locus on chromosome 6. Four of these genes (*PRF1, PRKRA, NLRP8*, and *HDAC7*) were novel and not in linkage disequilibrium (LD) with common variants identified from previous GWAS. These genes are involved in regulatory T-cell homeostasis and regulation, IFNγ biology, and NFκB signaling. Despite these novel findings, much of the heritability of MS remains unknown.

In the United States, approximately 5% of MS cases are diagnosed before age 18 years (pediatric-onset MS or “POMS”). POMS shares several risk factors with adult-onset MS, including HLA-DRB1*1501, the largest known genetic risk factor for MS.^6^ Studies of POMS cohorts may provide deeper insights into the genetics of MS because early age at onset may reflect higher genetic burden. Compared to adults, less is known about the contribution of genetic variants to POMS because of its low prevalence and the large sample size needed for genetic studies. To date, 28 non-MHC adult-onset MS GWAS variants have been implicated in POMS susceptibility.^6^ No studies have investigated whether rare or low-frequency variants are associated with POMS.

In this case–control study, we used an exome array to investigate the role of rare and low-frequency coding variants in 636 individuals with POMS and controls. Using a gene-based approach, we aimed to identify whether rare and low-frequency variants within the following genes were associated with POMS: four genes previously identified as having low-frequency variants associated with adult-onset MS (*PRF1, PRKRA, NLRP8*, and *HDAC7*) and genes within the MHC region.

## Materials and methods

### Study participants

POMS cases (*n* = 646) and controls (*n* = 498) were recruited from the US Network of Pediatric MS Centers, and 80 additional control subjects were recruited from Kaiser Permanente Northern California (KPNC), as previously described.^6,7^ Briefly, POMS cases were defined as individuals clinically diagnosed with MS onset or clinically isolated syndrome before age 18 years and were negative for antibodies against myelin oligodendrocyte glycoprotein. Control subjects were individuals without a diagnosis of MS or related condition (optic neuritis, transverse myelitis, or demyelination disease) recruited as part of a pediatric MS case–control study or recruited from Northern California. All study participants provided blood samples for DNA extraction. This study was approved by the local institutional review boards of all participating centers. All patients and parents signed assent and consent forms for this study.

### Genetic data

DNA from blood samples was genotyped using the Illumina OmniExpressExome BeadChip at the University of California, Berkeley. Subjects were excluded from analyses if they were missing more than 10% of genotypes, had sex discordance, or were related with a threshold of pihat > 0.2. Population stratification is a particular concern when studying rare genetic variation, and there currently lacks a well-established method that adjusts for population stratification in admixed populations. Therefore, the analytic sample was limited to individuals with ⩾90% European (CEU) ancestry which was estimated by single-nucleotide polymorphism (SNP) weights for various ancestral populations.^8^ This resulted in an analytic sample of 330 POMS cases and 306 controls.

Genotyped exome variants were removed if more than 10% of genotypes were missing in the sample; were in the pseudoautosomal region of X, chromosome Y, or mitochondrial genome; had no copies of the minor allele; or deviated from Hardy–Weinberg equilibrium among controls (*p* < 1 × 10^−4^). A total of 360,726 exome variants were analyzed. These were categorized into rare/low-frequency (MAF < 5% among controls) (*n* = 72,931) or common (MAF ⩾ 5% among controls) (*n* = 287,795) variants. For each subset, variants were assigned to genes using the UCSC Genome Browser. Variants not assigned to a gene were removed. Variants assigned to more than one gene were allocated to the gene with the greater number of assigned variants. Genes assigned less than five variants were excluded from analysis to improve statistical power.^9^ This resulted in 46,098 rare or low-frequency variants across 5078 genes and 133,822 common variants across 7176 genes for statistical analyses.

### Statistical analyses

To maximize power and reduce the burden of multiple comparisons with our modest sample size, we tested two specific hypotheses. First, whether four genes (*PRF1, PRKRA, NLRP8*, and *HDAC7*) containing low-frequency variants previously associated with adult-onset MS were associated with POMS. Second, whether genes containing rare and low-frequency variants within the MHC were associated with POMS. For each, we used a gene-based method called optimal sequence kernel association test (SKAT-O) which optimally combines gene-burden tests (best when variants are causal and have the same direction and magnitude of effect) and SKAT (best for variants with differing effects).^10,11^ Missing genotypes were imputed within the SKAT package using the “bestguess” method. All models adjusted for the first five principal components derived from principal components analysis of samples genotyped common variants.^12^ For each analysis, multiple comparisons corrections were performed using a Bonferroni-corrected threshold. Genes were considered significant if *p* < 0.05/number of genes tested.

We examined whether significant genes were specific to rare and low-frequency variants by repeating SKAT-O within significant genes but with common exome variants only. We also conducted a leave-one-out analysis of significant genes whereby one variant was iteratively removed and SKAT-O was rerun to determine whether specific rare and low-frequency variants were driving the significant effect.

All analyses were conducted using PLINK 1.9, R, and the SKAT package in R.^13,14^

## Results

Among 636 participants, 73% of POMS cases were female, while 68% of controls were female. The average age of MS onset was 14.16 (SD = 3.03) years.

Of the four genes harboring rare or low-frequency variants previously identified as associated with adult-onset MS, three (*PRF1, NLRP8*, and *HDAC7*) passed quality control and filtering steps to be included in analyses. Only *PRF1*, located on chromosome 10 and which included six rare or low-frequency coding variants, was associated with POMS (*p* = 2.70 × 10^−3^) (Table 1). The previously identified significant low-frequency, adult-onset MS coding variant at chr10:72360387 within *PRF1* had a MAF of 5.0% in the adult-onset cohort. This is similar to what we observed in POMS (MAF of 6.7% among cases and 3.1% among controls) (Supplemental Table 1). When this variant was removed using leave-one-out analyses and the SKAT-O test statistic was re-calculated, the gene was no longer significant (*p* = 0.44) (Supplemental Table 1). SKAT-O results from leave-one-out analyses of other variants within this gene did not result in similarly increased *p*-values.

**Table 1.** SKAT-O results for genes harboring rare or low-frequency variants previously identified as associated with adult-onset MS.

Chr	Gene	No. of variants	*p*-value
10	*PRF1*	6	2.70 × 10^−3^
12	*HDAC7*	8	0.38
19	*NLRP8*	14	0.77
2	*PRKRA*	0	NA

Chr: chromosome; No.: number; SKAT-O: optimal sequence kernel association test.

*PRKRA* not included in SKAT-O analyses because there were no rare/low-frequency variants within this gene in our dataset.

Although we did not determine that *HDAC7* was significantly associated with POMS in this study, the rare variant previously identified as associated with adult-onset MS within *HDAC7* (chr12:48191247) had a similar MAF among POMS cases (1.5%) compared to previously reported adult-onset MS cases and controls (1.4%).^4^ The MAF among POMS controls was 2.0%. There were no POMS cases or controls in our data with any copies of the minor allele for the significant adult-onset MS rare coding variant within *NLRP8*, chr19:56487619 (MAF = 0.2% in adult-onset MS cases and controls). No rare or low-frequency variants within *PRKRA* were present in our dataset. Mitrovič et al.^4^ reported the low-frequency variants previously identified as associated with adult-onset MS within *PRKRA* (chr2:179315031 and chr2:179315726) had a MAF 5.6% each among adult-onset MS cases and controls. In our sample, the MAFs among POMS cases were 5.15% and 5.03%, respectively, and 5.72% and 5.61%, respectively, among controls. Therefore, they were filtered out of our analyses for having a MAF > 5% among controls.

**Figure 1.**
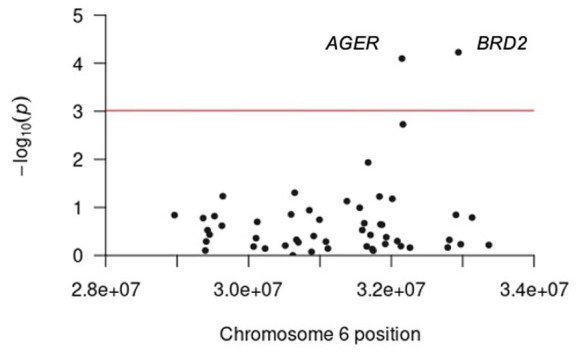
Two genes from SKAT-O analysis of rare and low-frequency variants within the MHC region were significantly associated with POMS. The line represents the Bonferroni significance cut-off (*p* = 0.05/52 MHC genes in the dataset). Genes were plotted at their starting location on the genome.

Of 52 MHC genes harboring rare or low-frequency variants tested in SKAT-O analyses, two were significant at *p* < 0.05/52 (Figure 1). These were *BRD2* (*p* *=* 5.89 × 10^−5^) and *AGER* (*p* *=* 7.96 × 10^−5^), which contained 13 and 9 rare/low-frequency variants, respectively (Supplemental Table 1). Of the 22 rare/low-frequency coding variants within *BRD2* and *AGER*, 21 were available in the 1000 Genomes CEU reference genome, and none were in LD with HLA-DRB1*1501. For 18 of these variants, the MAF was larger in controls than POMS cases, indicating minor alleles were protective of POMS (Supplemental Table 1). The largest difference in MAF among cases and controls for *BRD2* variants was from exm537239, which had a MAF of 4.77% among controls and 1.91% among POMS cases. The largest absolute difference in MAF among cases and controls for *AGER* variants was from rs2070600, with a MAF of 4.25% among controls and 1.78% among POMS cases. Leave-one-out analyses for variants within these genes did not result in *p*-values substantially different from those that included all variants, indicating single variants were not driving the observed gene effects in POMS.

We sought to determine whether the effects of *PRF1, BRD2*, and *AGER* were specific to rare/low-frequency variants or might be indicative of the effects of common variants by rerunning SKAT-O using common variants from each respective gene. There were not at least five common exome variants assigned to *PRF1* or *AGER*, so this could not be formally assessed. For *BRD2*, SKAT-O analyses of common variants were not significantly associated with POMS.

## Discussion

In this case–control study, we found evidence for the association of rare and low-frequency coding variants with POMS. These included variants within *PRF1*, a gene that was previously identified as associated with adult-onset MS, and two genes in the MHC region (*BRD2* and *AGER*). To the best of our knowledge, *BRD2* and *AGER* have not been identified previously as associated with POMS risk, and the effects of all three genes are independent of common variant signals. These genes would not have been identified by common variant studies and GWAS, emphasizing the merits of investigating rare genetic variation in complex diseases.

Our results confirm previous findings from an adult-onset MS study that *PRF1* is associated with MS risk. In the previous study, the low-frequency coding variant rs35947132, specifically, was associated with adult-onset MS. This variant was included in the set of rare/low frequency variants in our gene-based analysis of *PRF1* and is not in LD with other adult MS GWAS variants. Our study does not provide evidence for common coding variants within *PRF1* being associated with POMS, suggesting the effect of this gene might be specific to its rare/low-frequency variants.^4^ *PRF1* encodes perforin, a pore-forming protein that plays a key role in secretory granule-dependent cell death and in defense against virus-infected or neoplastic cells.^15–17^ Previous targeted studies have shown that variants within *PRF1* are associated with MS, and it has been demonstrated that human autoreactive CD4+ T cells specific for myelin basic protein mediate cytotoxicity using perforin pathways for target cell lysis.^18–20^ The agreement of our findings with those from adult-onset MS further emphasize the need for further studies to understand this pathway’s potential role in MS pathogenesis.

In addition to replicating the *PRF1* finding, we identified two novel genes in the MHC, *BRD2* and *AGER*, not previously associated with POMS. *BRD2*, an MHC class II gene, encodes bromodomain and extra-terminal domain (BET) proteins. BETs regulate the expression of many proinflammatory and immunoregulatory genes and pathways through their involvement in the epigenetic modification of histone acetylation.^21^ Our results identified that the rare, minor alleles within *BRD2* were protective for POMS. This might indicate that there exist uncommon changes to BETs that reduce risk of POMS, which aligns with past research showing that inhibiting BET decreases inflammation and demyelination in the EAE mouse model.^21,22^ Our other finding, *AGER*, encodes the receptor for advanced glycosylation end product (RAGE) which can induce a pro-inflammatory state through interaction with its ligands.^23^ One previous study of 168 MS patients and 136 controls genotyped three variants within *AGER* and found that the AA genotype at the −372 T/A polymorphism was significantly higher in the control group compared to MS patients.^24^ These results are consistent with the protective effect of rare variants within this gene that we observed for POMS. Interestingly, one small study of MS patients found that 12-month treatment with fingolimod increased serum levels of soluble RAGE isoforms, which are known to inhibit RAGE signaling, and decreased the rate of clinical relapse.^25^ At least some of the beneficial effect of fingolimod may be through modulation of the RAGE axis. Given that the rare minor alleles of *AGER* were also protective for POMS, future studies should investigate the functional and/or structural changes that result from these rare mutations within *AGER* to help better understand MS pathogenesis and potentially identify new drug targets for MS.

The current study had several strengths. While POMS is rare, we utilized the largest available clinically well-characterized cohort. We also confirmed that findings were not driven by LD with HLA-DRB1*1501, although other genes within the MHC might be causal variants. However, confounding of observed associations by patterns of LD, a substantial concern in common variant studies, is less likely for rare variants because they do not show substantial patterns of LD with other variants. This study also has several limitations. POMS is difficult to study due to its very low prevalence. This makes it challenging to conduct exome- or genome-wide studies due to power limitations. Our approach was to determine a priori genes/regions for investigation, which might have excluded important coding regions relevant to POMS susceptibility that remain to be discovered in larger studies. Additionally, we restricted analyses to individuals with European ancestry to reduce the effects of population stratification. This means the results of this study may not be generalizable to non-European populations. It also means rare variants that might be associated with POMS in other populations and provide useful clues for MS etiology were not identified. Additionally, our use of the Illumina exome BeadChip and gene-based analysis excluded rare and low-frequency variants in non-coding regions. Extending the current work to non-coding regions will be a challenge but should be strongly considered given the vast number of these regions across the genome are known to influence gene regulation.

In conclusion, this study provides evidence that rare and low-frequency coding variants associated with adult-onset MS (*PRF1*) and within the MHC region (*BRD2* and *AGER*) are associated with POMS susceptibility. These genes have not previously been identified by GWAS, emphasizing the merits of investigating rare genetic variation in MS. Given that coding variants encode protein sequences which are relatively easier to interpret and study than the effects of non-coding variation, future investigation of these genes and larger rare variant studies may provide helpful understanding of the pathobiology of MS.

## Supplemental Material

sj-xlsx-1-msj-10.1177_13524585221150736 – Supplemental material for Rare and low-frequency coding genetic variants contribute to pediatric-onset multiple sclerosis Supplemental material, sj-xlsx-1-msj-10.1177_13524585221150736 for Rare and low-frequency coding genetic variants contribute to pediatric-onset multiple sclerosis by Mary K Horton, Joan E Shim, Amelia Wallace, Jennifer S Graves, Gregory Aaen, Benjamin Greenberg, Soe Mar, Yolanda Wheeler, Bianca Weinstock-Guttman, Amy Waldman, Teri Schreiner, Moses Rodriguez, Jan-Mendelt Tillema, Tanuja Chitnis, Lauren Krupp, T Charles Casper, Mary Rensel, Janace Hart, Hong L Quach, Diana L Quach, Catherine Schaefer, Emmanuelle Waubant and Lisa F Barcellos in Multiple Sclerosis Journal
